# Osteosarcopenia predicts poor survival in patients with cirrhosis: a retrospective study

**DOI:** 10.1186/s12876-023-02835-y

**Published:** 2023-06-05

**Authors:** Chisato Saeki, Tomoya Kanai, Kaoru Ueda, Masanori Nakano, Tsunekazu Oikawa, Yuichi Torisu, Masayuki Saruta, Akihito Tsubota

**Affiliations:** 1 Division of Gastroenterology and Hepatology, Department of Internal Medicine, The Jikei University School of Medicine, Minato-Ku, Tokyo, Japan; 2 Division of Gastroenterology, Department of Internal Medicine, Fuji City General Hospital, Fuji-Shi, Shizuoka, Japan; 3 Project Research Units, Research Center for Medical Science, The Jikei University School of Medicine, Minato-Ku, Tokyo, Japan

**Keywords:** Cirrhosis, Prognosis, Osteosarcopenia

## Abstract

**Background:**

Osteosarcopenia, defined as the coexistence of sarcopenia and osteoporosis, is associated with adverse clinical outcomes. The present study investigated the prognostic significance of osteosarcopenia in patients with cirrhosis.

**Methods:**

This retrospective study evaluated 126 patients with cirrhosis. Participants were classified into three groups based on the presence or absence of (1) sarcopenia and/or osteoporosis; and (2) Child–Pugh (CP) class B/C cirrhosis and/or osteosarcopenia, and the cumulative survival rates were compared between the groups.

Cox proportional hazards model was used to identify independent factors associated with mortality. Sarcopenia and osteoporosis were diagnosed according to the Japan Society of Hepatology and the World Health Organization criteria, respectively.

**Results:**

Among the 126 patients, 24 (19.0%) had osteosarcopenia. Multivariate analysis identified osteosarcopenia as a significant and independent prognostic factor. The cumulative survival rates were significantly lower in patients with osteosarcopenia than in those without (1/3/5-year survival rates = 95.8%/73.7%/68.0% vs. 100%/93.6%/86.5%, respectively; *p* = 0.020). Patients with osteosarcopenia, but not sarcopenia or osteoporosis alone, had significantly lower cumulative survival rates than those without both conditions (*p* = 0.019). Furthermore, patients with both CP class B/C and osteosarcopenia had significantly lower cumulative survival rates than those without both (*p* < 0.001) and with either condition (*p* < 0.001).

**Conclusions:**

Osteosarcopenia was significantly associated with mortality in patients with cirrhosis. The cumulative survival rates were lower in patients with osteosarcopenia than in those without both conditions. Additionally, comorbid osteosarcopenia worsened the prognosis of patients with CP class B/C. Therefore, simultaneous evaluation of both sarcopenia and osteoporosis is crucial to better predict the prognosis.

**Supplementary Information:**

The online version contains supplementary material available at 10.1186/s12876-023-02835-y.

## Background

Sarcopenia, characterized by progressive decreases in skeletal muscle mass and function, is recognized as a frequent and serious complication in patients with cirrhosis [1]. In 2016, the Japan Society of Hepatology (JSH) proposed practical criteria for sarcopenia in chronic liver disease (CLD) [2]. Protein-energy malnutrition, hyperammonemia, decreased levels of anabolic hormones (e.g., insulin-like growth factor 1) and branched-chain amino acids, and elevated levels of inflammatory cytokines cause an imbalance between protein synthesis and proteolysis, leading to sarcopenia [2, 3]. Recent studies have shown that sarcopenia increases the risk of cirrhosis-related complications, such as ascites, infections, hepatic encephalopathy, and mortality [4–7]. A meta-analysis of 22 studies confirmed that sarcopenia increases the mortality risk by approximately 2-fold in patients with cirrhosis [8].

Osteoporosis, characterized by loss of bone mass and deterioration of bone microarchitecture, also is a common complication in patients with cirrhosis [9]. Similar to the pathogenesis of sarcopenia, malnutrition, dysregulation of the receptor activator of nuclear factor kappa-B (NF-κB) ligand (RANKL)/RANK/osteoprotegerin system due to chronic inflammation, and decreased levels of anabolic hormone and 25-hydroxyvitamin D cause an imbalance in the activities of osteoblasts and osteoclasts, leading to osteoporosis [10]. Osteoporosis and associated major osteoporotic fractures increase the mortality risk in the general population [11]. However, a meta-analysis of eight studies revealed that effective osteoporosis treatments can reduce the mortality rate by approximately 10% in older frail individuals who have a high risk of fracture [12]. Therefore, early and appropriate assessment and therapeutic intervention are crucial from a prognostic standpoint. Although the relationship between osteoporosis and mortality remains to be determined, patients with cirrhosis have a significantly higher risk of post-fracture complications, such as sepsis, acute renal failure, and 30-day in-hospital mortality [13]. Therefore, evaluating muscle and bone diseases together may be crucial to better predict liver disease-related events and prognosis in patients with cirrhosis.

Muscles and bones are closely interrelated during growth and maintenance by common factors, such as genetic factors, myokines, and osteokines [3]. Consequently, sarcopenia and osteoporosis often develop or progress simultaneously, and the term osteosarcopenia was coined in 2009 to define the coexistence of these comorbidities [14]. Osteosarcopenia is associated with malnutrition and aggravates the risk of falls, fractures, and impaired physical performance in older adults, leading to reduced quality of life and poor prognosis [15–21]. The prevalence of osteosarcopenia is reportedly 16.8% in patients with chronic liver disease (CLD) [22]. This condition has a negative impact on the clinical outcomes of patients, such as an increased prevalence of vertebral fractures and frailty [22]. Despite the well-known relationship between osteosarcopenia and mortality in the general population, only one report has evaluated this relationship in patients with CLD [23]. However, it was limited to only patients who underwent hepatic resection for hepatocellular carcinoma (HCC) and did not assess handgrip strength, which is a fundamental component of the diagnosis criteria for sarcopenia. In the present study, we diagnosed sarcopenia following the JSH criteria (including the assessment of handgrip strength) [2] and aimed to investigate the impact of osteosarcopenia on the prognosis of patients with cirrhosis who had no HCC.

## Methods

### Participants

In this retrospective study, we analyzed 126 consecutive patients with cirrhosis who visited Fuji City General Hospital between 2017 and 2020. This study cohort included 118 patients analyzed in our previous report [9]. The inclusion criteria were (1) presence of cirrhosis diagnosed on the basis of laboratory tests and endoscopic/imaging findings, such as the presence of esophageal/gastric varices, ascites, liver deformations, and surface irregularities; and (2) consent to undergo muscle and bone measurement tests. The exclusion criteria were (1) pre-existence of malignant diseases, including hepatocellular carcinoma (HCC);　and (2) presence of a pacemaker, implants, or refractory ascites. Serum albumin, total bilirubin, aspartate aminotransferase (AST), alanine aminotransferase (ALT), Mac-2 binding protein glycosylation isomer (M2BPGi, a hepatic fibrosis marker), platelet, and prothrombin time–international normalized ratio (PT–INR) were measured using standard laboratory methods. The fibrosis-4 (FIB-4) index was calculated using the following formula: FIB-4 = age (years) × AST (U/L)/(platelet count [10^9^ /L] × ALT^1/2^ [U/L]) [24]. Liver functional reserve was evaluated using the Child–Pugh (CP) classification and model for end-stage liver disease (MELD) score [25, 26]. HCC was diagnosed according to the HCC guidelines of the American Association for the Study of Liver Diseases [27]. Patients who underwent liver transplantation during the observation period were considered to be dead and censored cases.

### Diagnosis of sarcopenia, osteoporosis, and osteosarcopenia

Sarcopenia was diagnosed according to the revised criteria proposed by the Japan Society of Hepatology (second edition) [28]. In brief, sarcopenia is defined as skeletal muscle mass index (SMI) < 7.0 kg/m^2^ for men and < 5.7 kg/m^2^ for women, measured using bioimpedance analysis (InBody S10; InBody, Seoul, Korea), and handgrip strength < 28 kg for men and < 18 kg for women, measured using a dynamometer (T.K.K5401 GRIP-D; Takei Scientific Instruments, Niigata, Japan). Gait speed was measured over a distance of 6 m, and slow gait speed was defined as < 0.8 m/s [29]. Osteoporosis was diagnosed using the World Health Organization (WHO) criteria (T-score ≤  − 2.5) [30]. The bone mineral density (BMD) of the lumbar spine (L2–L4), femoral neck, and total hip was assessed using dual-energy X-ray absorptiometry (PRODIGY; GE Healthcare Japan, Tokyo, Japan). At least one of these three measurements met the WHO criteria in the diagnosis of osteoporosis. Osteosarcopenia was defined as the coexistence of sarcopenia and osteoporosis [14].

### Statistical analysis

Continuous and categorical variables are presented as median (interquartile range) and number (percentage), respectively. Between-group differences were assessed using the Mann–Whitney U test for continuous variables and the chi-squared test for categorical variables. Correlations between BMD and SMI or handgrip strength were analyzed using the Spearman's rank correlation test. The cumulative survival rates were estimated using the Kaplan–Meier method and were compared between the groups using the log-rank test and the Bonferroni multiple comparisons method. Significant and independent factors associated with mortality were identified using univariate and multiple Cox proportional hazards models. SPSS Statistics version 27 (IBM Japan, Tokyo, Japan) was used for all statistical analyses. Values of *p* < 0.05 were considered statistically significant.

## Results

### Study population and characteristics

A flow diagram of patients included in this study is shown in Figure S1. Of the 156 patients with cirrhosis evaluated for eligibility, 30 met the exclusion criteria and the remaining 126 patients were enrolled. Table 1 presents the baseline characteristics of the participants. This study cohort included 77 (61.1%) men, with a median age of 70. 5 (57.8–76.0) years. The median MELD score was 9.0 (7.0–12.0). The rate of CP class B or C (B/C) was 34.9% (44/126).

**Table 1:** Comparison of clinical characteristics between patients with and without osteosarcopenia

Variable	All patients	Osteosarcopenia	Non-osteosarcopenia	*p-*value
Patients, n (%)	126	24 (19.0)	102 (81.0)	
Men, n (%)	77 (61.1)	10 (41.7)	67 (65.7)	0.030
Age (years)	70.5 (57.8–76.0)	76.0 (73.0–80.0)	68.0 (56.5–75.0)	0.001
BMI (kg/m^2^)	23.6 (21.2–25.8)	20.7 (18.7–22.2)	24.1 (22.2–26.2)	< 0.001
Etiology				
HBV/HCV/Alcohol/other, n	13/36/47/30	1/12/4/7	12/24/43/23	0.022
Child–Pugh B/C, n (%)	44 (34.9)	6 (25.0)	38 (37.3)	0.257
MELD score	9.0 (7.0–12.0)	8.0 (7.0–10.0)	9.0 (7.0–12.0)	0.603
Total bilirubin (mg/dL)	0.9 (0.6–1.3)	0.8 (0.5–1.2)	0.9 (0.7–1.3)	0.212
Albumin (g/dL)	3.9 (3.4–4.3)	4.0 (3.3–4.4)	3.8 (3.5–4.2)	0.791
Prothrombin time INR	1.11 (1.04–1.22)	1.07 (1.01–1.13)	1.14 (1.04–1.27)	0.030
FIB-4	4.16 (2.76–5.57)	4.75 (3.27–5.47)	3.87 (2.74–5.69)	0.441
M2BPGi (C.O.I)	2.97 (1.47–5.60)	2.59 (1.98–3.54)	3.32 (1.39–6.13)	0.558
SMI (kg/m^2^)				
All patients	6.76 (5.87–7.52)	5.22 (4.58–5.64)	7.04 (6.33–7.82)	< 0.001
Men	7.30 (6.65–8.09)	5.63 (5.25–6.20)	7.38 (6.96–8.16)	< 0.001
Women	5.87 (5.07–6.45)	4.78 (4.49–5.23)	6.08 (5.81–6.58)	< 0.001
Handgrip strength (kg)				
All patients	24.5 (16.7–31.9)	14.5 (12.6–18.0)	28.3 (21.0–34.5)	< 0.001
Men	30.7 (24.9–36.7)	20.5 (13.0–24.9)	31.6 (28.1–37.4)	< 0.001
Women	16.5 (13.8–21.7)	14.3 (10.1–15.7)	19.1 (14.6–22.5)	< 0.001
Slow gait speed, n (%)	36 (28.6)	15 (62.5)	21 (20.6)	< 0.001
Lumbar spine BMD (kg/m^2^)	1.09 (0.90–1.23)	0.90 (0.83–0.99)	1.16 (0.95–1.26)	< 0.001
Femoral neck BMD (kg/m^2^)	0.79 (0.65–0.90)	0.60 (0.53–0.65)	0.83 (0.72–0.93)	< 0.001
Total hip BMD (kg/m^2^)	0.83 (0.71–0.96)	0.63 (0.55–0.71)	0.87 (0.77–0.98)	< 0.001
BCAA supplementation, n (%)	25 (19.8)	2 (8.3)	23 (22.5)	0.116

Continuous variables are shown as median (interquartile range). Statistical analysis was performed using the chi-squared test or the Mann–Whitney U test, as appropriate. *BCAA* branched-chain amino acid, *BMD* bone mineral density, *BMI* body mass index, *C.O.I* cut-off index, *FIB-4* fibrosis-4, *HBV* hepatitis B virus, *HCV* hepatitis C virus, *INR* international normalized ratio, *M2BPGi* Mac-2 binding protein glycosylation isomer, *MELD* model for end-stage liver disease, *SMI* skeletal muscle mass index

### Clinical characteristics of patients with and without osteosarcopenia

The prevalence of osteosarcopenia was 19.0% (24/126; Table 1). Men accounted for 41.7% and 65.7% of patients with and without osteosarcopenia, respectively, with osteosarcopenia being more frequent in women (*p* = 0.030). Patients with osteosarcopenia were significantly older (*p* = 0.001) and had lower body mass index (BMI) (*p* < 0.001) and higher prevalence of slow gait speed (*p* < 0.001) than those without osteosarcopenia. Regarding laboratory data, patients with osteosarcopenia had lower PT–INR levels than those without (*p* = 0.030). The most frequent etiologies were hepatitis C virus in the osteosarcopenia group and alcohol in the non-osteosarcopenia group.

### Correlations between BMD and SMI or handgrip strength

The SMI and handgrip strength were significantly correlated with BMD at lumbar spine (*r* = 0.378 and 0.291; *p* < 0.001 and = 0.010, respectively), femoral neck (*r* = 0.547 and 0.458; *p* < 0.001 for both, respectively), and total hip (*r* = 0.519 and 0.402; *p* < 0.001 for both, respectively) in men (Figure S2A–S2F). Similarly, these sarcopenia-related factors were significantly correlated with BMD at lumbar spine (*r* = 0.427 and 0.413; *p* = 0.002 and = 0.003, respectively), femoral neck (*r* = 0.442 and 0.527; *p* = 0.001 and < 0.001, respectively), and total hip (*r* = 0.490 and 0.520; *p* < 0.001 for both, respectively) in women (Figure S3A–S3F).

### Prognostic factors in patients with cirrhosis

The median observation period was 57.1 (44.2–61.6) months, during which 16.7% (21/126) of the patients died from liver disease-related events (liver failure, *n* = 12; liver transplantation, *n* = 1; HCC, *n* = 3; and rupture of esophageal varices, *n* = 5) (Figure S1). Univariate analysis revealed that BMI, CP class B/C, MELD score, sarcopenia, osteoporosis, and osteosarcopenia were significantly associated with mortality (Table S1). Cox proportional hazards regression analysis identified CP class B/C (hazard ratio [HR], 7.045; 95% confidence interval [CI], 2.692–18.441; *p* < 0.001) osteosarcopenia (HR, 4.798; 95%CI, 1.885–12.212; *p* = 0.001) as significant and independent prognostic factors in patients with cirrhosis (Table 2).

**Table 2:** Significant factors associated with mortality in patients with liver cirrhosis

	Univariate		Multivariate	
Variable	HR (95%CI)	*p*-value	HR (95%CI)	*p*-value
BMI (kg/m^2^)	0.894 (0.795–1.005)	0.062		
Child–Pugh B/C	4.821 (1.938–11.996)	< 0.001	7.045 (2.692–18.441)	< 0.001
MELD score	1.187 (1.043–1.351)	0.009		
Sarcopenia	2.595 (1.101–6.120)	0.029		
Osteoporosis	2.616 (1.099–6.226)	0.030		
Osteosarcopenia	2.752 (1.133–6.683)	0.025	4.798 (1.885–12.212)	0.001

*BMI* body mass index, *CI* confidence interval, *HR* hazard ratio, *MELD* model for end-stage liver disease

### Impact of sarcopenia, osteoporosis, and osteosarcopenia on survival

**Fig. 1:**
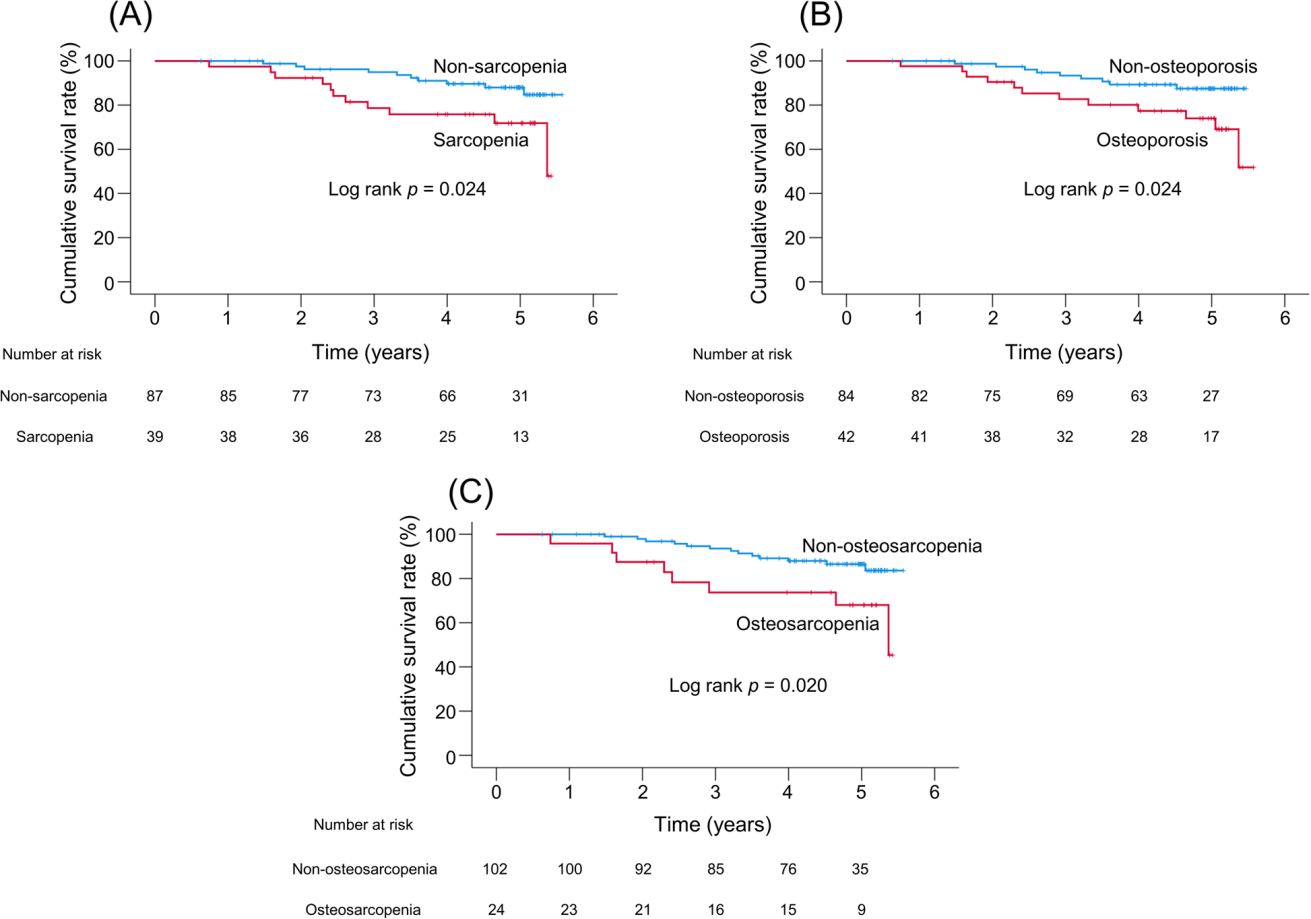
Cumulative survival rates in patients with and without sarcopenia (**A**), osteoporosis (**B**), and osteosarcopenia (**C**)

The 1-, 3-, and 5-year cumulative survival rates were 97.4% vs. 100%, 78.6% vs. 94.9%, and 71.8% vs. 88.0% in the sarcopenia and non-sarcopenia groups, respectively, showing they were significantly lower in the sarcopenia group than in the non-sarcopenia group (*p* = 0.024, Fig. 1A).

The cumulative survival rates were significantly lower in the osteoporosis group than in the non-osteoporosis group (*p* = 0.024, Fig. 1B). The 1-, 3-, and 5-year cumulative survival rates were 97.6% vs. 100%, 82.7% vs. 93.4%, and 74.0% vs. 87.5% in the osteoporosis group and non-osteoporosis group, respectively.

Regarding the difference in prognoses, the cumulative survival rates were significantly lower in the osteosarcopenia group than in the non-osteosarcopenia group (*p* = 0.020, Fig. 1C). The 1-, 3-, and 5-year cumulative survival rates were 95.8% vs. 100%, 73.7% vs. 93.6%, and 68.0% vs. 86.5% in the osteosarcopenia and non-osteosarcopenia groups, respectively.

**Fig. 2:**
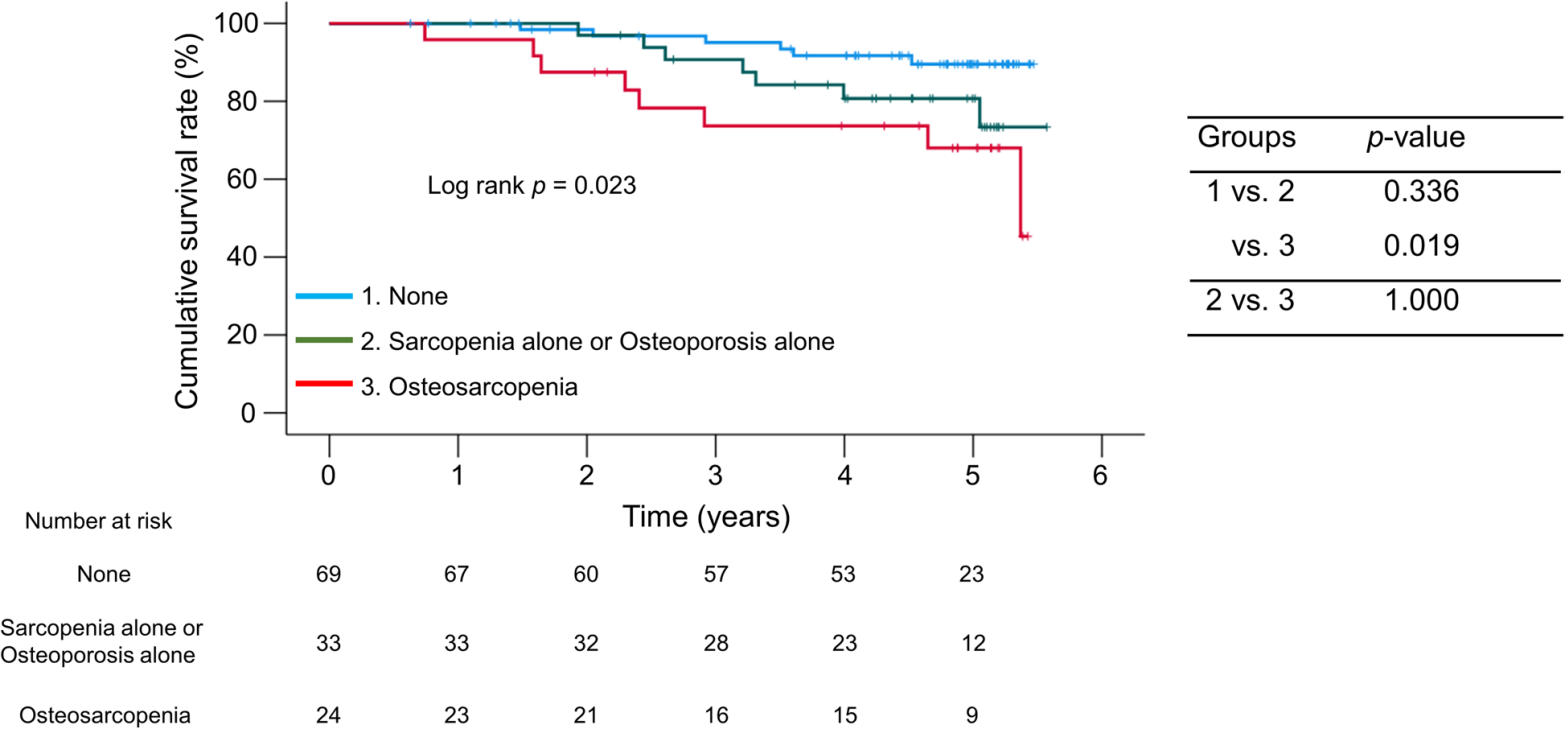
Prognostic impact of osteosarcopenia on patients with cirrhosis. Patients with osteosarcopenia had significantly lower survival rates than those without both sarcopenia and osteoporosis (*p* = 0.019)

On the basis of the presence or absence of sarcopenia and/or osteoporosis, we classified the patients into three groups: (i) patients without both sarcopenia and osteoporosis (*n* = 69), (ii) patients with sarcopenia alone or osteoporosis alone (*n* = 33), and (iii) patients with osteosarcopenia (n = 24). Patients with osteosarcopenia (but not either condition alone) had significantly lower cumulative survival rates than those without both conditions (*p* = 0.019) (Fig. 2).

### Impact of comorbidity of CP class B/C cirrhosis and osteosarcopenia on survival

**Fig. 3:**
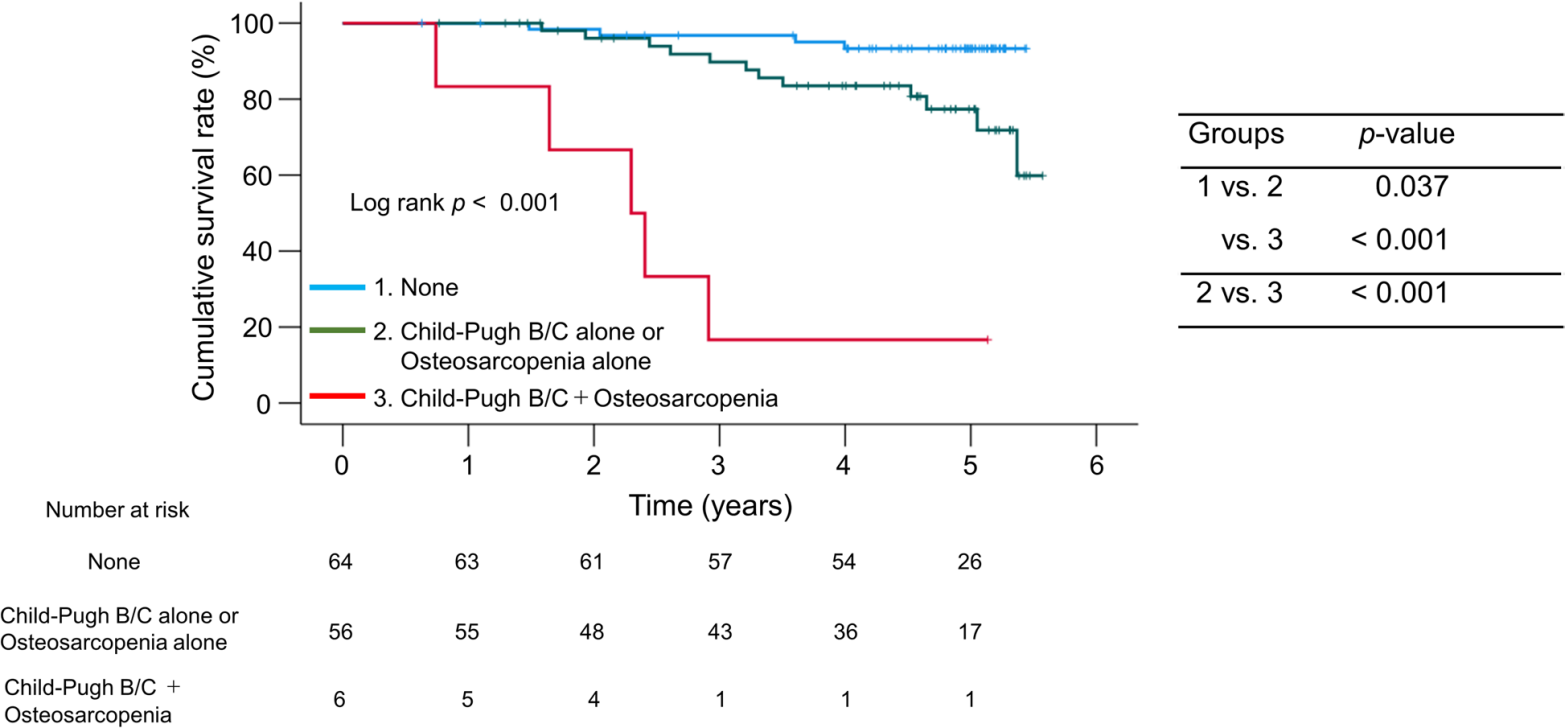
Prognostic impact of the coexistence of Child–Pugh class B/C cirrhosis and osteosarcopenia. The cumulative survival rates were significantly lower for patients with either or both conditions than for those without both conditions (*p* = 0.037 or < 0.001, respectively). There was a significant difference in the cumulative survival rates between patients with either and both conditions (*p* < 0.001)

We classified the patients into three groups based on the presence or absence of CP class B/C cirrhosis and/or osteosarcopenia: (i) patients without both CP class B/C and osteosarcopenia (*n* = 64), (ii) patients with either condition alone (*n* = 56), and (iii) patients with both conditions (*n* = 6). Patients with both conditions had significantly lower cumulative survival rates than those without both (*p* < 0.001) and with either condition alone (*p* < 0.001) (Fig. 3).

## Discussion

Sarcopenia and osteoporosis are closely interrelated with each other and often develop or progress simultaneously (i.e., osteosarcopenia) [9]. Indeed, bone mass was significantly correlated with muscle mass or muscle strength in both men and women in this study. Osteosarcopenia increases the risk of falls, fractures, and mortality, leading to reduced quality of life and adverse outcomes [15–21]. As such, this condition has attracted a great deal of attention in recent years. This is the first study to focus on the relationship between osteosarcopenia and prognosis of cirrhotic patients without HCC. In this study, multivariate analysis identified osteosarcopenia as a significant and independent factor associated with mortality. Of note, patients with osteosarcopenia had lower survival rates than those without both conditions. Additionally, comorbid osteosarcopenia worsened the prognosis of patients with CP class B/C.

In one study of community-dwelling older adults, mortality was significantly higher in participants with osteosarcopenia (HR, 2.48) than in those without [17]. However, neither sarcopenia nor osteoporosis alone were significantly associated with increased mortality, similar to the results of the present study. In another study of patients with hip fracture, the 1-year mortality rate was higher (15.1%; HR, 1.84) for patients with osteosarcopenia than for those without osteosarcopenia (without both sarcopenia and osteoporosis, 7.8%; with sarcopenia alone, 10.3%; and with osteoporosis alone, 5.1%) [16]. Furthermore, osteosarcopenia was reported to be one of the strongest prognostic factors for patients who underwent hepatic resection for HCC, intrahepatic cholangiocarcinoma, and colorectal liver metastases [23, 31, 32]. A pooled analysis of three cohort studies, including 2,601 participants, revealed that osteosarcopenia significantly increased the mortality risk, with an odds ratio of 1.66 [18]. These findings indicate that osteosarcopenia is associated with poor prognosis, irrespective of underlying diseases or conditions, and emphasize that osteosarcopenia is more critical in predicting patient prognosis than sarcopenia or osteoporosis alone. Therefore, it is critically important to evaluate both sarcopenia and osteoporosis together in patients with cirrhosis.

Patients with cirrhosis frequently have malnutrition due to reduced energy and protein intake, malabsorption, altered nutrient metabolism, hormonal imbalance, hypermetabolism, and inflammation, leading to secondary sarcopenia and osteoporosis [33, 34]. Reportedly, nutritional status, as assessed by the mini nutritional assessment- short form, was worse in patients with osteosarcopenia than in those with sarcopenia or osteoporosis alone [20]. Malnourished patients had a significantly higher risk of cirrhosis-related complications requiring hospitalization (such as ascites, infections, and hepatic encephalopathy), sarcopenia, and mortality than well-nourished patients [35]. The coexistence of sarcopenia and osteoporosis aggravates physical performance, increasing the risk of falls, fractures, and frailty [15, 17–21]. Patients with lower physical performance and frailty were reported to be at higher risk for cirrhosis-related complications, hospital readmission, and mortality [36]. Collectively, patients with osteosarcopenia are more likely to develop liver disease-related events and worsen prognosis due to malnutrition and impaired physical performance.

The CP classification is widely used to evaluate liver functional reserve. This scoring system is a good predictor of prognosis in patients with cirrhosis; i.e., survival rates are lower for patients with CP class B/C than for those with CP class A [37]. In this study, patients with CP class B/C complicated by osteosarcopenia had the lowest survival rates among the three stratified groups. A previous study showed that the presence of sarcopenia reduced the cumulative survival rates in patients with CP class A/B [38]. Additionally, comorbid sarcopenia worsened the 2-year survival rates in patients with CP class C or MELD score > 14 [39]. Therefore, it is conceivable that combining osteosarcopenia with CP class B/C will identify patients with the most serious prognosis.

Given that osteosarcopenia is associated with poor prognosis, early and comprehensive therapeutic intervention is crucial for longevity. Reportedly, long-term branched-chain amino acid (BCAA) administration significantly improved muscle mass, muscle strength, and muscle function, and mortality in sarcopenic patients with cirrhosis [40, 41]. In this study, the rate of BCAA supplementation in the osteosarcopenia group was extremely low, suggesting the need for earlier nutritional intervention. In one study of CLD patients with osteoporosis, administration of denosumab, a human monoclonal antibody for RANKL, significantly improved BMD and bone quality marker (i.e., plasma pentosidine) [42]. Intriguingly, the 5-year denosumab treatment improved sarcopenia-related parameters (such as muscle strength and physical performance) as well as BMD in older adults [43]. In another study of older men with osteosarcopenia, high-intensity resistance training with vitamin D, calcium, and protein supplementation increased the SMI and maintained BMD [44]. Despite the importance of these treatments, early and appropriate assessment and intervention for osteosarcopenia remain undetermined in real-world clinical settings. In the future, treatment strategies for sarcopenia, osteoporosis, and osteosarcopenia should be established in patients with CLD from a prognostic perspective.

This study had some limitations. First, we did not investigate the patients’ nutritional intake or daily activities, which might have influenced the muscle and bone mass measurements. Second, patients with refractory ascites, who might be more susceptible to osteosarcopenia, were excluded due to the unreliability of the bioimpedance analysis.

Finally, the sample size was not large enough to evaluate the impact of osteosarcopenia on the patient prognosis.

## Conclusions

In conclusion, this study demonstrated that osteosarcopenia increases the mortality risk in patients with cirrhosis. Therefore, early and appropriate assessment and intervention for both sarcopenia and osteoporosis are crucial to improve the patient prognosis.

## Supplementary Information

**Additional file 1: Figure S1.** Flow diagram of patients included in this study.

**Additional file 2: Figure S2.** Correlations between the skeletal muscle mass indexor handgrip strength and bone mineral densityof the lumbar spine, femoral neck, and total hip in men. The SMI was significantly correlated with the BMD of the lumbar spine, femoral neck, and total hip. The handgrip strength was significantly correlated with the BMD of the lumbar spine, femoral neck, and total hip.

**Additional file 3: Figure S3.** Correlations between the skeletal muscle mass indexor handgrip strength and bone mineral densityof the lumbar spine, femoral neck, and total hip in women. The SMI was significantly correlated with the BMD of the lumbar spine, femoral neck, and total hip. The handgrip strength was significantly correlated with the BMD of the lumbar spine, femoral neck, and total hip.

**Additional file 4: Table S1.** Univariate analysis of factors associated with mortality.

## Data Availability

The datasets used and analyzed during the current study are available from the corresponding author on reasonable request.

## Abbreviations

CI: Confidence interval
CLD: Chronic liver disease
CP: Child–Pugh
HCC: Hepatocellular carcinoma
HR: Hazard ratio
MELD: Model for end-stage liver disease
PT–INR: Prothrombin time–international normalized ratio
RANK: Receptor activator of nuclear factor kappa-B
SMI: Skeletal muscle mass index

## References

[1] Yoshiji H, Nagoshi S, Akahane T, Asaoka Y, Ueno Y, Ogawa K (2021). Evidence-based clinical practice guidelines for liver cirrhosis 2020. Hepatol Res.

[2] Nishikawa H, Shiraki M, Hiramatsu A, Moriya K, Hino K, Nishiguchi S (2016). Japan Society of Hepatology guidelines for sarcopenia in liver disease (1st edition): Recommendation from the working group for creation of sarcopenia assessment criteria. Hepatol Res.

[3] Saeki C, Tsubota A (2021). Influencing factors and molecular pathogenesis of sarcopenia and osteosarcopenia in chronic liver disease. Life (Basel).

[4] Kim G, Kang SH, Kim MY, Baik SK (2017). Prognostic value of sarcopenia in patients with liver cirrhosis: a systematic review and meta-analysis. PLoS One.

[5] Wijarnpreecha K, Werlang M, Panjawatanan P, Kroner PT, Cheungpasitporn W, Lukens FJ (2020). Association between sarcopenia and hepatic encephalopathy: a systematic review and meta-analysis. Ann Hepatol.

[6] Kikuchi N, Uojima H, Hidaka H, Iwasaki S, Wada N, Kubota K (2021). Prospective study for an independent predictor of prognosis in liver cirrhosis based on the new sarcopenia criteria produced by the Japan Society of Hepatology. Hepatol Res.

[7] Zeng X, Shi ZW, Yu JJ, Wang LF, Luo YY, Jin SM (2021). Sarcopenia as a prognostic predictor of liver cirrhosis: a multicentre study in China. J Cachexia Sarcopenia Muscle.

[8] Tantai X, Liu Y, Yeo YH, Praktiknjo M, Mauro E, Hamaguchi Y (2022). Effect of sarcopenia on survival in patients with cirrhosis: a meta-analysis. J Hepatol.

[9] Saeki C, Takano K, Oikawa T, Aoki Y, Kanai T, Takakura K (2019). Comparative assessment of sarcopenia using the JSH, AWGS, and EWGSOP2 criteria and the relationship between sarcopenia, osteoporosis, and osteosarcopenia in patients with liver cirrhosis. BMC Musculoskelet Disord.

[10] Jeong HM, Kim DJ (2019). Bone diseases in patients with chronic liver disease. Int J Mol Sci.

[11] Shiraki M, Kuroda T, Tanaka S (2011). Established osteoporosis associated with high mortality after adjustment for age and co-mobidities in postmenopausal Japanese women. Intern Med.

[12] Bolland MJ, Grey AB, Gamble GD, Reid IR (2010). Effect of osteoporosis treatment on mortality: a meta-analysis. J Clin Endocrinol Metab.

[13] Chen TL, Lin CS, Shih CC, Huang YF, Yeh CC, Wu CH (2017). Risk and adverse outcomes of fractures in patients with liver cirrhosis: two nationwide retrospective cohort studies. BMJ Open.

[14] Binkley N, Buehring B (2009). Beyond FRAX: it's time to consider "sarco-osteopenia". J Clin Densitom.

[15] Kirk B, Zanker J, Duque G (2020). Osteosarcopenia: epidemiology, diagnosis, and treatment-facts and numbers. J Cachexia Sarcopenia Muscle.

[16] Yoo JI, Kim H, Ha YC, Kwon HB, Koo KH (2018). Osteosarcopenia in Patients with Hip Fracture Is Related with High Mortality. J Korean Med Sci.

[17] Salech F, Marquez C, Lera L, Angel B, Saguez R, Albala C (2021). Osteosarcopenia predicts falls, fractures, and mortality in Chilean community-dwelling older adults. J Am Med Dir Assoc.

[18] Teng Z, Zhu Y, Teng Y, Long Q, Hao Q, Yu X (2021). The analysis of osteosarcopenia as a risk factor for fractures, mortality, and falls. Osteoporos Int.

[19] Sepúlveda-Loyola W, Phu S, Bani Hassan E, Brennan-Olsen SL, Zanker J, Vogrin S (2020). The joint occurrence of osteoporosis and sarcopenia (Osteosarcopenia): definitions and characteristics. J Am Med Dir Assoc.

[20] Reiss J, Iglseder B, Alzner R, Mayr-Pirker B, Pirich C, Kässmann H (2019). Sarcopenia and osteoporosis are interrelated in geriatric inpatients. Z Gerontol Geriatr.

[21] Okayama A, Nakayama N, Kashiwa K, Horinouchi Y, Fukusaki H, Nakamura H (2022). Prevalence of sarcopenia and its association with quality of life, postural stability, and past incidence of falls in postmenopausal women with osteoporosis: a cross-sectional study. Healthcare (Basel).

[22] Saeki C, Kanai T, Nakano M, Oikawa T, Torisu Y, Abo M (2020). Relationship between osteosarcopenia and frailty in patients with chronic liver disease. J Clin Med.

[23] Yanagaki M, Haruki K, Taniai T, Igarashi Y, Yasuda J, Furukawa K, et al. The significance of osteosarcopenia as a predictor of the long-term outcomes in hepatocellular carcinoma after hepatic resection. J Hepatobiliary Pancreat Sci 2022 Online ahead of print. 10.1002/jhbp.1246 36181339

[24] Sterling RK, Lissen E, Clumeck N, Sola R, Correa MC, Montaner J (2006). Development of a simple noninvasive index to predict significant fibrosis in patients with HIV/HCV coinfection. Hepatology.

[25] Malinchoc M, Kamath PS, Gordon FD, Peine CJ, Rank J, ter Borg PC (2000). A model to predict poor survival in patients undergoing transjugular intrahepatic portosystemic shunts. Hepatology.

[26] Pugh RN, Murray-Lyon IM, Dawson JL, Pietroni MC, Williams R (1973). Transection of the oesophagus for bleeding oesophageal varices. Br J Surg.

[27] Marrero JA, Kulik LM, Sirlin CB, Zhu AX, Finn RS, Abecassis MM (2018). Diagnosis, staging, and management of hepatocellular carcinoma: 2018 practice guidance by the American Association for the study of liver diseases. Hepatology.

[28] Nishikawa H, Shiraki M, Hiramatsu A, Hara N, Moriya K, Hino K (2021). Reduced handgrip strength predicts poorer survival in chronic liver diseases: a large multicenter study in Japan. Hepatol Res.

[29] Cruz-Jentoft AJ, Bahat G, Bauer J, Boirie Y, Bruyère O, Cederholm T (2019). Sarcopenia: revised European consensus on definition and diagnosis. Age Ageing.

[30] WHO (1994). Assessment of fracture risk and its application to screening for postmenopausal osteoporosis. WHO Study Group. World Health Organ Tech Rep Ser.

[31] Taniai T, Haruki K, Yanagaki M, Igarashi Y, Furukawa K, Onda S, et al. Osteosarcopenia predicts poor prognosis for patients with intrahepatic cholangiocarcinoma after hepatic resection. Surg Today. 2022 Online ahead of print. 10.1007/s00595-022-02550-3 35831486

[32] Furukawa K, Haruki K, Taniai T, Hamura R, Shirai Y, Yasuda J (2021). Osteosarcopenia is a potential predictor for the prognosis of patients who underwent hepatic resection for colorectal liver metastases. Ann Gastroenterol Surg.

[33] Traub J, Reiss L, Aliwa B, Stadlbauer V (2021). Malnutrition in patients with liver cirrhosis. Nutrients.

[34] Walker-Bone K (2012). Recognizing and treating secondary osteoporosis. Nat Rev Rheumatol.

[35] Maharshi S, Sharma BC, Srivastava S (2015). Malnutrition in cirrhosis increases morbidity and mortality. J Gastroenterol Hepatol.

[36] Essam Behiry M, Mogawer S, Yamany A, Rakha M, Awad R, Emad N (2019). Ability of the short physical performance battery frailty index to predict mortality and hospital readmission in patients with liver cirrhosis. Int J Hepatol.

[37] D'Amico G, Garcia-Tsao G, Pagliaro L (2006). Natural history and prognostic indicators of survival in cirrhosis: a systematic review of 118 studies. J Hepatol.

[38] Kang SH, Jeong WK, Baik SK, Cha SH, Kim MY (2018). Impact of sarcopenia on prognostic value of cirrhosis: going beyond the hepatic venous pressure gradient and MELD score. J Cachexia Sarcopenia Muscle.

[39] Zeng X, Shi ZW, Yu JJ, Wang LF, Luo YY, Jin SM (2021). Sarcopenia as a prognostic predictor of liver cirrhosis: a multicentre study in China. J Cachexia Sarcopenia M uscle.

[40] Singh Tejavath A, Mathur A, Nathiya D, Singh P, Raj P, Suman S (2021). Impact of branched chain amino acid on muscle mass, muscle strength, physical performance, combined survival, and maintenance of liver function changes in laboratory and prognostic markers on sarcopenic patients with liver cirrhosis (BCAAS Study): a randomized clinical trial. Front Nutr.

[41] Hanai T, Shiraki M, Nishimura K, Ohnishi S, Imai K, Suetsugu A (2015). Sarcopenia impairs prognosis of patients with liver cirrhosis. Nutrition.

[42] Saeki C, Saito M, Oikawa T, Nakano M, Torisu Y, Saruta M (2020). Effects of denosumab treatment in chronic liver disease patients with osteoporosis. World J Gastroenterol.

[43] Miedany YE, Gaafary ME, Toth M, Hegazi MO, Aroussy NE, Hassan W (2021). Is there a potential dual effect of denosumab for treatment of osteoporosis and sarcopenia?. Clin Rheumatol.

[44] Kemmler W, Kohl M, Fröhlich M, Jakob F, Engelke K, von Stengel S (2020). Effects of high-intensity resistance training on osteopenia and sarcopenia parameters in older men with osteosarcopenia-one-year results of the randomized controlled Franconian Osteopenia and Sarcopenia Trial (FrOST). J Bone Miner Res.

