# Supplementary figures and images for: Osteosarcopenia predicts poor survival in patients with cirrhosis: a retrospective study
Source: BMC Gastroenterol. 2023 Jun 5;23:196. doi: 10.1186/s12876-023-02835-y (PMC10242914; doi:10.1186/s12876-023-02835-y)

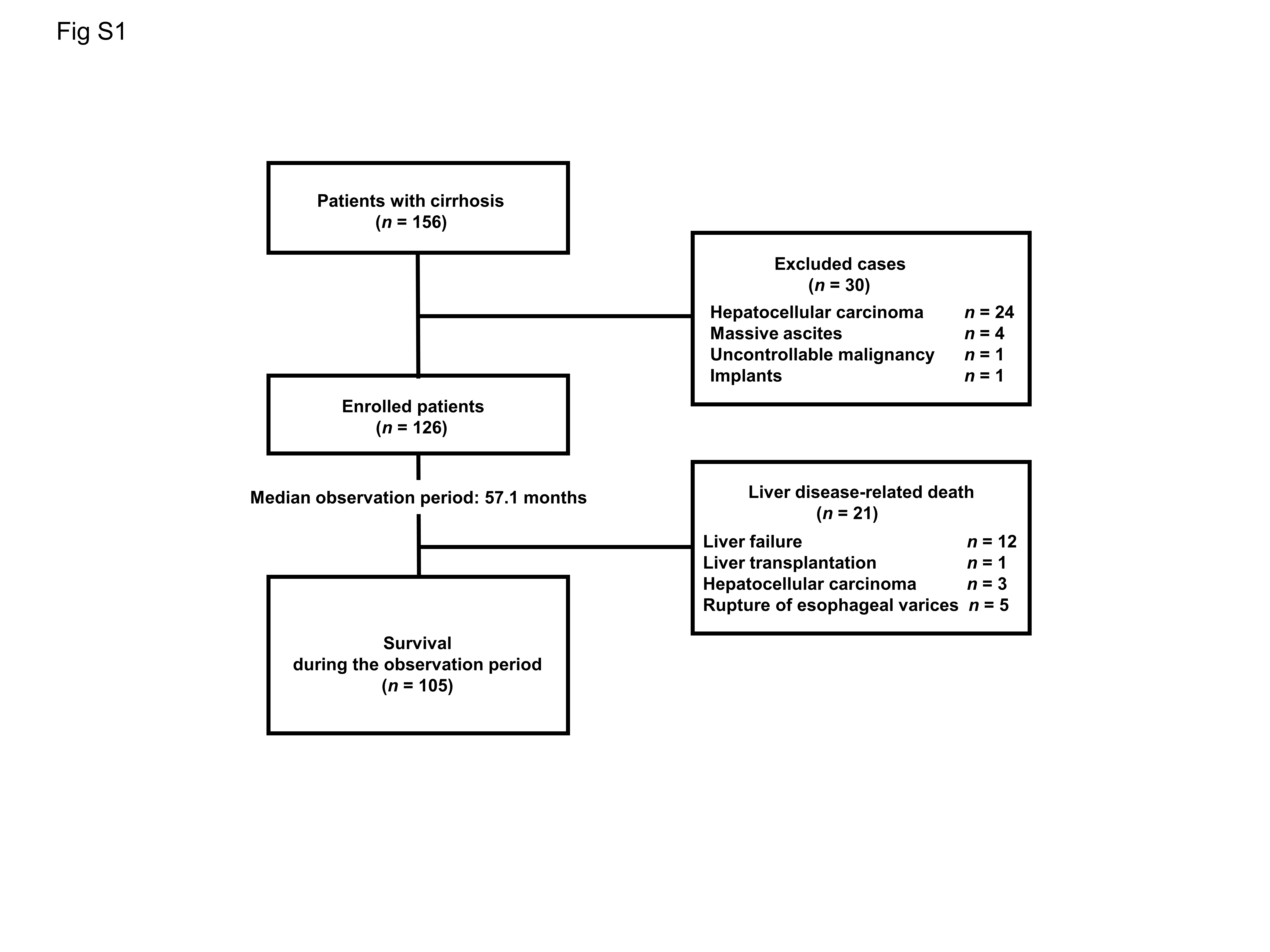

Supplement: Supplementary file 1 — Additional file 1: Figure S1. Flow diagram of patients included in this study. [file 12876_2023_2835_MOESM1_ESM.tif]

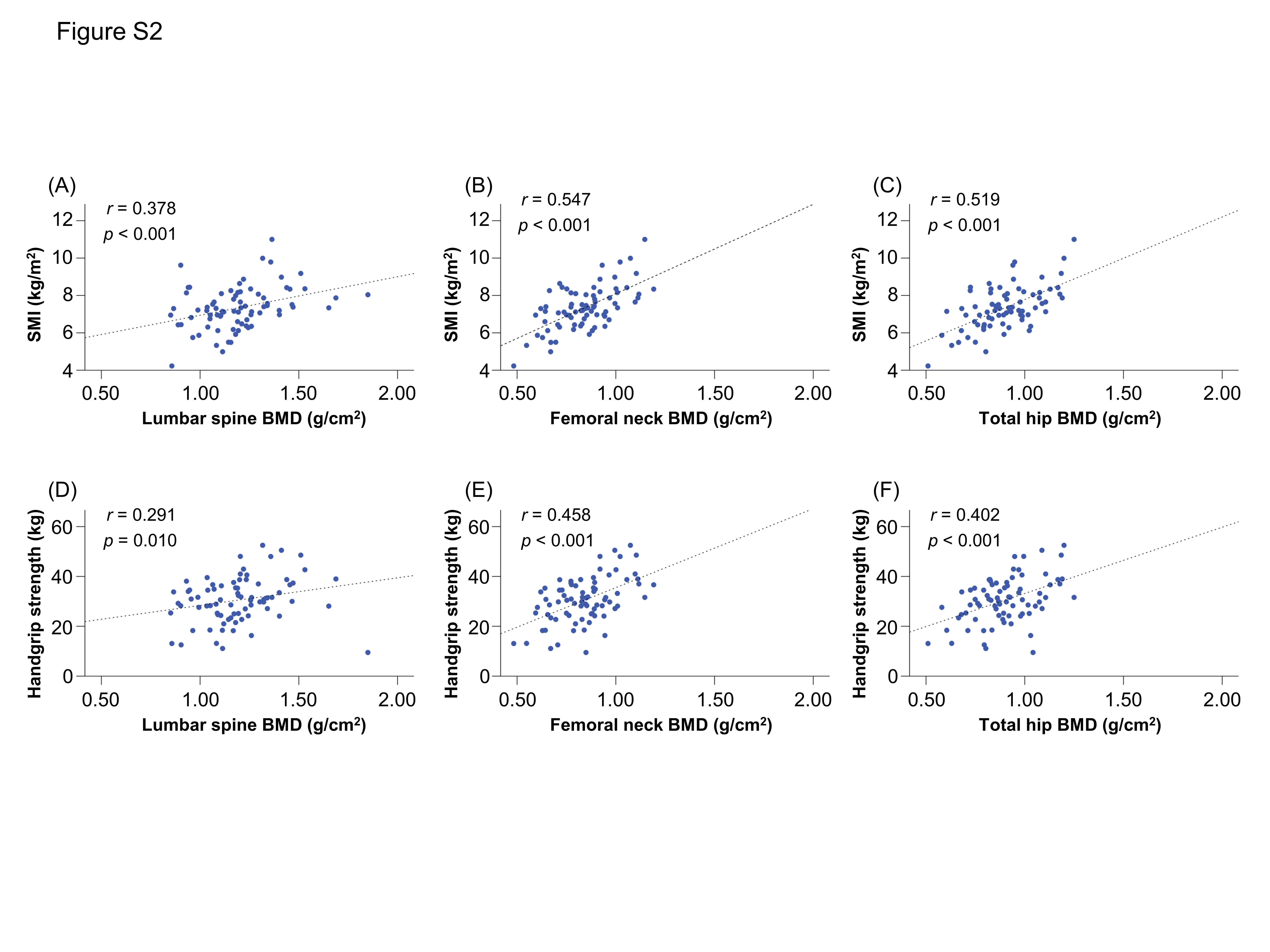

Supplement: Supplementary file 2 — Additional file 2: Figure S2. Correlations between the skeletal muscle mass indexor handgrip strength and bone mineral densityof the lumbar spine, femoral neck, and total hip in men. The SMI was significantly correlated with the BMD of the lumbar spine, femoral neck, and total hip. The handgrip strength was significantly correlated with the BMD of the lumbar spine, femoral neck, and total hip. [file 12876_2023_2835_MOESM2_ESM.tif]

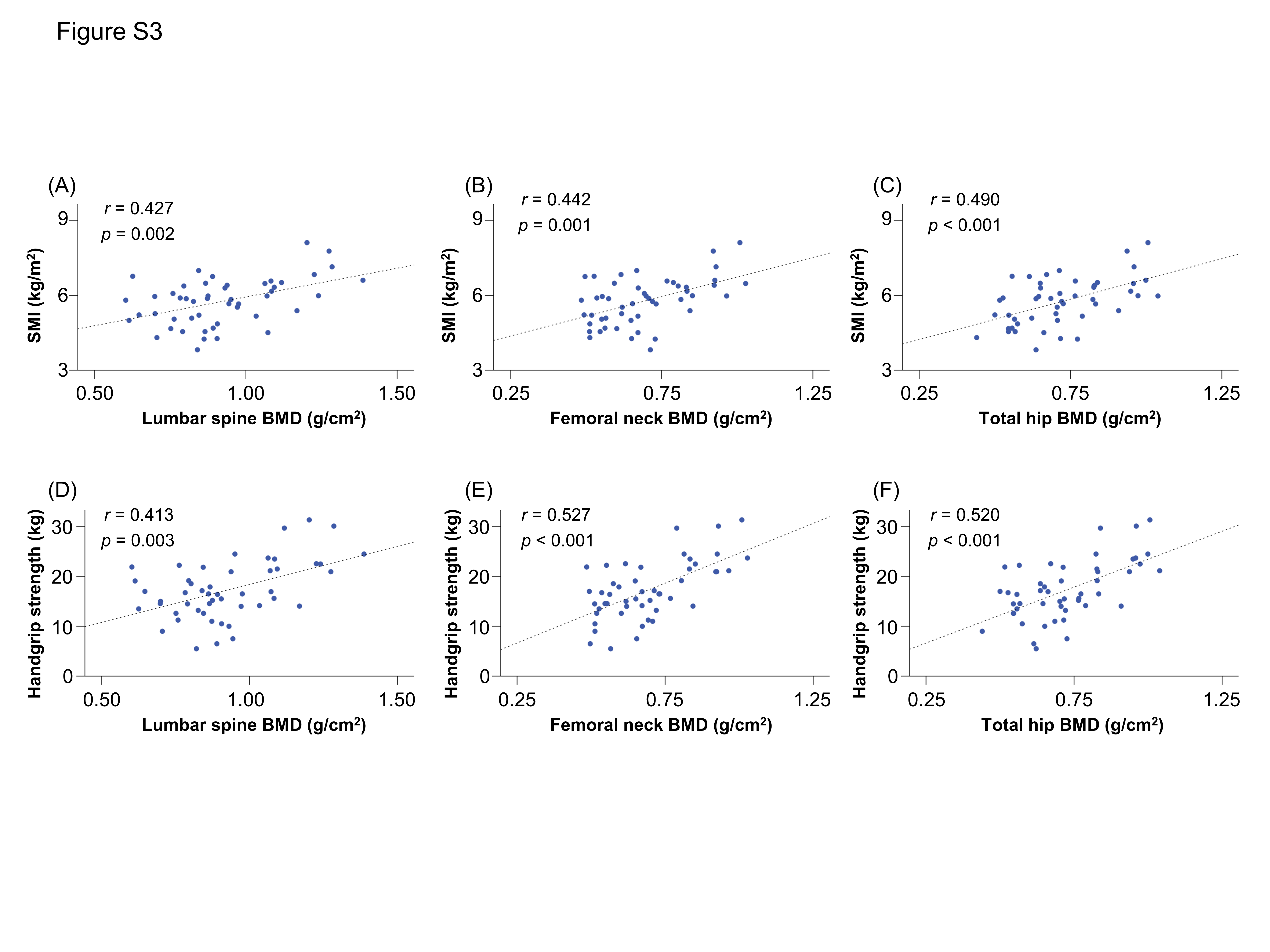

Supplement: Supplementary file 3 — Additional file 3: Figure S3. Correlations between the skeletal muscle mass indexor handgrip strength and bone mineral densityof the lumbar spine, femoral neck, and total hip in women. The SMI was significantly correlated with the BMD of the lumbar spine, femoral neck, and total hip. The handgrip strength was significantly correlated with the BMD of the lumbar spine, femoral neck, and total hip. [file 12876_2023_2835_MOESM3_ESM.tif]
