# Supplementary material for: Osteosarcopenia predicts poor survival in patients with cirrhosis: a retrospective study
Source: BMC Gastroenterol. 2023 Jun 5;23:196. doi: 10.1186/s12876-023-02835-y (PMC10242914; doi:10.1186/s12876-023-02835-y)
Supplement: Supplementary file 4 — Additional file 4: Table S1. Univariate analysis of factors associated with mortality. [file 12876_2023_2835_MOESM4_ESM.docx]

**Table S1. Univariate analysis of factors associated with mortality**

| Variable | HR (95% CI) | *p*-value |
| --- | --- | --- |
| Gender (Men) | 1.081 (0.448–2.608) | 0.863 |
| Age (years) | 1.001 (0.964–1.039) | 0.966 |
| BMI (kg/m^2^) | 0.894 (0.795–1.005) | 0.062 |
| Etiology | 1.259 (0.780–2.031) | 0.345 |
| Child-Pugh B/C | 4.821 (1.938–11.996) | < 0.001 |
| MELD score | 1.187 (1.043–1.351) | 0.009 |
| FIB-4 | 1.098 (0.982–1.229) | 0.101 |
| M2BPGi (C.O.I) | 1.041 (0.964–1.124) | 0.311 |
| Sarcopenia | 2.595 (1.101–6.120) | 0.029 |
| Osteoporosis | 2.616 (1.099–6.226) | 0.030 |
| Osteosarcopenia | 2.752 (1.133–6.683) | 0.025 |

BMI, body mass index; CI, confidence interval; C.O.I, cut-off index; FIB-4, fibrosis-4; HR, hazard ratio; M2BPGi, Mac-2 binding protein glycosylation isomer; MELD, model for end-stage liver disease.
